# High level production of tyrosinase in recombinant *Escherichia coli*

**DOI:** 10.1186/1472-6750-13-18

**Published:** 2013-02-27

**Authors:** Qun Ren, Bernhard Henes, Michael Fairhead, Linda Thöny-Meyer

**Affiliations:** 1 Laboratory for Biomaterials, Swiss Federal Laboratories for Materials Science and Technology (Empa), Lerchenfeldstrasse 5, CH-9014 St, Gallen, Switzerland

**Keywords:** Tyrosinase, Recombinant protein production, High cell density, Fed batch culture, Bioprocess engineering, Exponential feeding

## Abstract

**Background:**

Tyrosinase is a bifunctional enzyme that catalyzes both the hydroxylation of monophenols to *o*-diphenols (monophenolase activity) and the subsequent oxidation of the diphenols to *o*-quinones (diphenolase activity). Due to the potential applications of tyrosinase in biotechnology, in particular in biocatalysis and for biosensors, it is desirable to develop a suitable low-cost process for efficient production of this enzyme. So far, the best production yield reported for tyrosinase was about 1 g L^-1^, which was achieved by cultivating the filamentous fungus *Trichoderma reesei* for 6 days*.*

**Results:**

In this work, tyrosinase from *Verrucomicrobium spinosum* was expressed in *Escherichia coli* and its production was studied in both batch and fed-batch cultivations. Effects of various key cultivation parameters on tyrosinase production were first examined in batch cultures to identify optimal conditions. It was found that a culture temperature of 32 °C and induction at the late growth stage were favorable, leading to a highest tyrosinase activity of 0.76 U mL^-1^. The fed-batch process was performed by using an exponential feeding strategy to achieve high cell density. With the fed-batch process, a final biomass concentration of 37 g L^-1^ (based on optical density) and a tyrosinase activity of 13 U mL^-1^ were obtained in 28 hours, leading to a yield of active tyrosinase of about 3 g L^-1^. The highest overall volumetric productivity of 103 mg of active tyrosinase per liter and hour (corresponding to 464 mU L^-1^ h^-1^) was determined, which is approximately 15 times higher than that obtained in batch cultures.

**Conclusions:**

We have successfully expressed and produced gram quantities per liter of active tyrosinase in recombinant *E. coli* by optimizing the expression conditions and fed-batch cultivation strategy. Exponential feed of substrate helped to prolong the exponential phase of growth, to reduce the fermentation time and thus the cost. A specific tyrosinase production rate of 103 mg L^−1^ h^−1^ and a maximum volumetric activity of 464 mU L^−1^ h^-1^ were achieved in this study. These levels have not been reported previously.

## Background

Tyrosinase is a bifunctional enzyme that catalyzes both the hydroxylation of monophenols to *o*-diphenols (monophenolase activity) and the subsequent oxidation of the diphenols to *o*-quinones (diphenolase activity) [1]. Tyrosinase is essential for many living organisms to carry out various functions, including melanin biosynthesis as defense against the harmful effects of UV light [2-4]. In plants, it is required for the biosynthesis of phenolic polymers such as lignin, flavinoids, and tannins [5]. Tyrosinases also play an important role in the regulation of the oxidation-reduction potential of cell respiration and in wound healing in plants [6,7].

Due to the ability of tyrosinases to react with phenols, these enzymes have been proposed for the uses in a variety of biotechnological, biosensor and biocatalysis applications [1,8,9]. For example, tyrosinases can be applied in detoxification of phenol-containing wastewater and contaminant soils [10], synthesis of L-3,4-dihydroxyphenylalanine (L-DOPA), one of the preferred drugs for the treatment of Parkinson‘s disease [11], or as additives in food processes due to their cross-linking abilities [12,13]. Tailoring polymers, e.g. grafting of silk proteins onto chitosan via tyrosinase reactions have also been reported [14,15]. Immobilized tyrosinase has been investigated as an electrochemical biosensor for a range of phenolic compounds [16]. The enzyme can react with exposed tyrosyl side chains in polypeptides, and the reactive quinones formed allow for protein-protein cross-linking [17-21].

Tyrosinases have been isolated and purified from various sources such as animals, plants, fungi, and bacteria [2,22-25]. The commercial production of tyrosinase is mostly reported from the common mushroom *Agaricus bisporus*. Extensive research has been carried out by using this mushroom tyrosinase because of its commercial availability. However, the use of tyrosinase from this source is problematic as the enzyme exhibits relatively low solvent and temperature stability, as compared to some bacterial tyrosinases [26-28]. Moreover, commercial tyrosinases are typically contaminated with other enzymes for example different isoforms, resulting in preparations of variable quality and activity [29]. Almost all reported tyrosinase-producing microbial (both fungal and bacterial) strains also produce other polyphenol oxidases such as peroxidase and laccase [30]. The presence of laccase and peroxidase along with tyrosinase imposes serious problems for commercial usage [31]. All these enzymes can use tyrosine as substrate but produce different products, resulting in reduced yield as well as increased cost for the downstream process. Recently, it was reported that a new *Actinomycetes* isolate produced only tyrosinase and did not exhibit peroxidase or laccase activities [30]. The production yield of tyrosinase was about 4.8 U mL^-1^ after 48 h incubation, which is still far too low to be applied for commercial purposes.

The recombinant production of tyrosinases becomes an attractive alternative to obtain large amounts of protein. Recombinant strains offer the possibility of a higher protein production level, better growth, and consequently, an improved productivity when compared to non-recombinant production systems. However, tyrosinases appear to be difficult to express in recombinant hosts and only a few examples have been reported. The human tyrosinase has been expressed mostly as insoluble protein in inclusion bodies in *Escherichia coli*[32,33]. Tyrosinase from *Pycnoporus sanguineus* was expressed in *Aspergillus niger*[34], and the *Streptomyces castaneoglobisporus* tyrosinase was expressed in a complex with its “caddy” protein, ORF378, in *E. coli*[35]. Tyrosinase production has also been reported for the filamentous fungus *Trichoderma reesei*[36]. Overexpression of the tyrosinase gene allowed the native host *T. reesei* to produce about 1 g L^-1^ tyrosinase in laboratory-scale batch fermentation after 6 days of cultivation [36]. Since growth of filamentous fungi is slow compared with most single-cell microorganisms, *Pichia pastoris* carrying the tyrosinase gene of *T. reesei* was tested and yielded 24 mg L^-1^ active recombinant tyrosinase in 3 days [37]. The *E. coli* recombinant was also used as host to produce tyrosinase from *Streptomyces sp.* REN-21, and 54 mg L^-1^ of the enzyme were obtained in the cytoplasm after 16 h incubation [26]. Recently, we have overexpressed and characterized the tyrosinase from *Verrucomicrobium spinosum* in *E. coli*[38]. However, even though the yield of this enzyme (150 mg L^-1^) was higher than those reported from other bacterial tyrosinases (unpublished data), we considered this yield could be further improved.

*E. coli* is commonly used as host for the rapid and economical production of recombinant proteins. Different cultivation techniques have been developed to increase the final biomass concentration [39,40]. However, high-level production of functional proteins in *E. coli* may not be a routine matter and is sometimes challenging. Not every protein can be produced efficiently due to the unique structural features of the protein, its folding pathways and its degradation by host cell proteases [41]. Since useful recombinant proteins have to be biologically active, the objectives for production should include not only maximization of the amount of recombinant protein, but also of total enzyme activity.

The current work is based on the successful cloning of the tyrosinase gene from *V. spinosum* in *E. coli* and its purification and characterization [38]. The objective of this work was to develop a suitable strategy for efficient production of active tyrosinase in *E. coli*. The effect of temperature, inducer isopropyl-beta-D-thiogalactopyranosides (IPTG) concentrations and the starting time of induction in different operation modes (batch and fed-batch) were investigated. About 3 g L^-1^ active tyrosinase were obtained after 28 hours of incubation under the developed conditions. To our knowledge, this is the best yield and productivity ever reported for recombinant tyrosinase.

## Results and discussion

### Production of tyrosinase in batch culture

In order to establish an efficient production process for tyrosinase, preliminary optimization studies were performed in shake flasks. This allowed the selection of the best production host, growth temperature, time for induction and inducer concentration by using conventional methodology with changing one variable at a time.

#### Influence of different host strains on tyrosinase production

To reach efficient tyrosinase production, cells with high growth rate, high maximum biomass and high level tyrosinase activity are desired. However, overproduction of a protein often hinders the growth of bacterial cells and *vice versa*[42]*.* Thus, it is plausible to let the cells grow to a high cell density, and then induce expression of the relevant gene so that the protein of interest will be produced. In this study, *E. coli* DH5α, JM109 and BLR were transformed with pMFvpt which contains the gene encoding the cytoplasmic full-length tyrosinase (53.5 kDa) of *Verrucomicrobium spinosum*[38]. The obtained recombinants were cultivated in shake flasks at 37°C and 150 rpm, and induced with 1 mM IPTG at the early exponential growth phase (OD_600_ ≈ 0.6). The cultivations were continued for 24 h and samples were taken periodically for measurement of cell density and tyrosinase activity. The results are summarized in Table 1. Although the DH5α recombinant exhibited the highest tyrosinase production, the growth rate was more than 2 fold lower than that obtained with two other tested hosts. Furthermore, the expression of the tyrosinase gene could not be controlled in recombinant DH5α because DH5α does not have the repressor protein LacI^q^ and enables constitutive expression of tyrosinase gene from the T5 promoter / *lac* operator element in pMFvpt. BLR exhibited uncontrolled expression and very low activity of tyrosinase. By contrast, the JM109 recombinant showed controlled expression due to the presence of the LacI^q^ repressor, even though the total tyrosinase production was lower than that in DH5α. Furthermore, the JM109 recombinant exhibited a high growth rate of 0.6 h^-1^. In order to reach high cell density, high growth rate and controlled production of the relevant protein are desired. Thus, in this study JM109 recombinant was chosen for further investigations.

**Table 1:** **Comparison of different *E. coli *hosts for tyrosinase production**

**Hosts **^**a**^	**μ**_**max **_**(h**^**-1**^**) **^**b**^	**OD**_**600max**_^**c**^	**Max. act. (U mL**^**-1**^**) **^**d**^
DH5α	0.26 ± 0.04	7.7 ± 0.4	0.44 ± 0.05
DH5α + IPTG	0.27 ± 0.03	8.0 ± 0.3	0.44 ± 0.01
BLR	0.60 ± 0.01	8.9 ± 0.4	0.03 ± 0.00
BLR + IPTG	0.57 ± 0.02	7.5 ± 0.6	0.03 ± 0.00
JM109	0.60 ± 0.02	7.4 ± 0.3	0.03 ± 0.00
JM109 + IPTG	0.59 ± 0.03	7.7 ± 0.4	0.07 ± 0.01

^a^. Cells were grown in shake flasks at 37°C with or without 1 mM IPTG induction at the early growth phase (OD_600_ ≈ 0.6).

^b^. μ_max_: the maximal growth rate.

^c^. OD_600max_: the maximal optical density measured at 600 nm.

^d^. Max. act.: the maximal volumetric activity of tyrosinase in units per mL of culture broth.

#### Influence of oxygen supply on tyrosinase production

**Figure 1:**
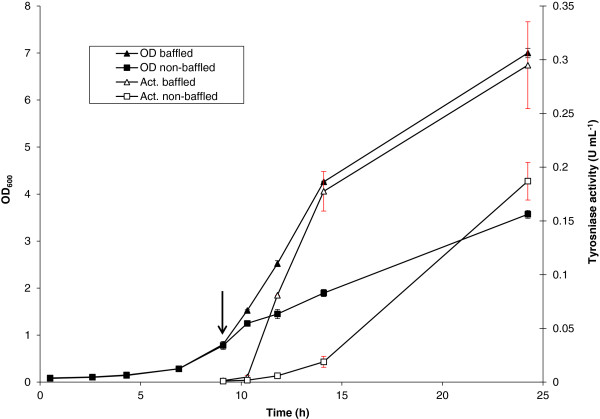
**Time course of cell growth and tyrosinase activity influenced by O**_**2**_**.** The freshly transformed *E. coli* JM109 (pMFvpt) recombinant cells were grown in shake flasks at 37°C and induced at the early exponential growth phase with 1.0 mM IPTG. The arrow indicates the time of IPTG induction. Data points are the averages of the results of duplicate measurements.

To investigate the influence of oxygen on tyrosinase production, experiments were conducted by comparing the cultures in baffled and non-baffled shake flasks. It was found that the total tyrosinase activity increased with an increase of cell density (Figure 1). Compared with the non-baffled culture, the baffled culture led to a higher final cell density and consequently to a higher total tyrosinase activity. Thus, sufficient oxygen supply is advantageous for tyrosinase production. These data demonstrated that tyrosinase production is associated with cell density: the volumetric tyrosinase activity increased in the same manner as the exponential growth of the cells (Figure 1).

#### Influence of induction stage on tyrosinase production

The influence of different concentrations of IPTG on cell growth and tyrosinase production was first tested. It was found that the biomass, the growth rate and the total tyrosinase activities were not influenced significantly by the tested IPTG concentrations between 0.1 mM and 1.0 mM (data not shown).

**Figure 2:**
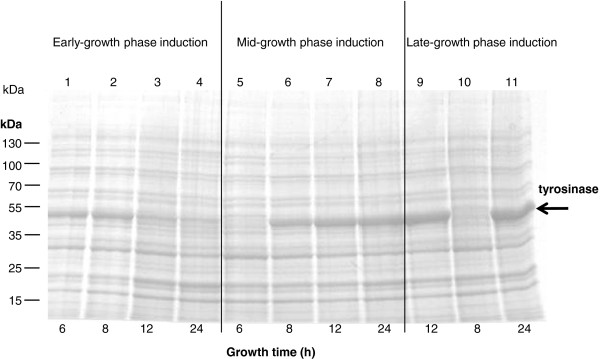
**Influence of induction stages on tyrosinase production.** The freshly transformed *E. coli* JM109 (pMFvpt) recombinant cells were grown in shake flasks at 37°C and induced with 1 mM IPTG at the early (from the beginning of the cultivation), mid (6 h cultivation) or late (8 h cultivation) growth phase. The extracts of *E. coli* JM109 (pMFvpt) were prepared and analyzed by SDS-PAGE. Lanes 1–4, samples from induction at the beginning of the cultivation; Lanes 5–8, samples from induction at mid-growth phase; lanes 9–11, samples from induction at late-growth phase. The arrow indicates the position of tyrosinase in the gel. Molecular weight standards are shown on the left.

As mentioned above, overexpression of a protein places an additional metabolic burden on the energy of the cells, carbon and amino acid pools, which may result in reduced cell growth. This can be avoided by employing inducible expression systems. The culture conditions at the time of induction can affect the efficiency of induction. In this study, IPTG induction was initiated at different growth stages, i.e. in the early-, mid- or late-exponential growth phase (Figure 2 and Table 2). Figure 2 shows that early induction led to immediate tyrosinase production within 6 h of cultivation. However, the tyrosinase band on the SDS-PAG disappeared after 12 h of cultivation, indicating the enzyme was either degraded or diluted with the increase of biomass. Induction at the mid-growth phase resulted in the production of tyrosinase after 2 h induction. The tyrosinase levels increased with cultivation time and reached the maximum at the end of cultivation (24 h) (Figure 2). Induction at the late-growth phase gave a high level of tyrosinase already after 4 h of induction (i.e. after 12 h of growth) and prolonged cultivation did not cause significant increase of the enzyme (Figure 2). The best stage for induction was thus the late-exponential growth phase: even though the growth rate and maximal biomass were similar to those of induction at mid-growth stage, the total tyrosinase activity was about 1.6 fold higher than in the latter case (Table 2). The early induction led to the lowest growth rate and lowest maximal biomass. The total tyrosinase activity was also 10–15 fold lower than that at mid- or late-growth phase induction (Table 2).

**Table 2:** Comparison of induction stages for tyrosinase production

**Growth stage **^**a**^	**μ**_**max **_**(h**^**-1**^**) **^**b**^	**OD**_**600max**_^**c**^	**Max. act. (U mL**^**-1**^**) **^**d**^
Early	0.39 ± 0.03	7.2 ± 0.5	0.05 ± 0.01
Mid	0.57 ± 0.02	8.4 ± 0.5	0.47 ± 0.07
Late	0.57 ± 0.03	8.9 ± 1.0	0.76 ± 0.06

^a^. Cells were induced with 1 mM IPTG at the early-, mid- or late- growth phase.

^b^. μ_max_: the maximal growth rate.

^c^. OD_600max_: the maximal optical density measured at 600 nm.

^d^. Max. act.: the maximal volumetric activity of tyrosinase in units per mL of culture broth.

JM109 (pMFvpt) cells were grown in shake flasks at 37°C.

Investigation of the induction stage is an important parameter for the development of the optimized protein production, especially when a strong promoter system is used, which was the case in this study. Induction of a strong promoter often leads to a sudden burst of protein synthesis which may inhibit cell growth due to a severe metabolic burden [43]. This can be well demonstrated by the results obtained here: early induction led to a reduced growth rate (Table 2).

#### Influence of temperature on tyrosinase production

For high cell density cultures, temperature control is very important due to considerable heat release at high viscosity. Temperature should support cell growth as well as product formation. In this study, time courses at temperatures of 25, 30, 32, and 37°C were followed. Decreasing the temperature from 37°C to 25°C led to decrease of grow rate, as expected. At 25°C only about half of the growth rate reached at 37°C was achieved (Table 3). The impact of the incubation temperature on tyrosinase production could also be clearly seen in Table 3. The best tyrosinase production (0.76 U mL^-1^) was observed at 32°C. An increase from 32°C to 37°C resulted in about 2 fold lower enzyme production, while the shift of incubation temperature from 32°C to 30°C reduced the enzyme production 3.5 fold (Table 3). Decrease of temperature from 32°C to 25°C caused a more than 5 fold reduction of the total enzyme yield. Time course experiments at different temperatures allowed to quickly optimize expression conditions for high level production of tyrosinase.

**Table 3:** Influence of culture temperature on tyrosinase production

**Temperature**	**μ**_**max **_**(h**^**-1**^**) **^**a**^	**OD**_**600max**_^**b**^	**Max. act. (U mL**^**-1**^**) **^**c**^
25 (°C)	0.31 ± 0.02	7.8 ± 0.9	0.12 ± 0.03
30 (°C)	0.52 ± 0.02	8.0 ± 0.7	0.22 ± 0.03
32 (°C)	0.60 ± 0.03	10.2 ± 0.9	0.76 ± 0.06
37 (°C)	0.66 ± 0.02	10.8 ± 1.0	0.36 ± 0.05

^a^. μ_max_: the maximal growth rate.

^b^. OD_600max_: the maximal optical density measured at 600 nm.

^c^. Max. act.: the maximal volumetric activity of tyrosinase in units per mL of culture broth.

The freshly transformed JM109 (pMFvpt) cells were grown in shake flasks at 25, 30, 32 or 37°C and induced at the early-exponential growth phase (OD_600_ ≈ 1.1) with 1.0 mM IPTG.

### Tyrosinase production using fed-batch cultivation

Different processes focusing on nutrient feeding strategies have been developed to grow cells to high cell densities and to overproduce proteins [44]. The most important function of such strategies is to prevent overfeeding, as inhibitory concentrations of the feed components can accumulate in the fermenter, or underfeeding, by which the organism is starved for essential nutrients. The method of choice depends on many different factors, including the metabolism of the organism, the potential for production of inhibitory substrates and induction conditions. Batch [45], continuous [46], and a variety of fed-batch processes [39,47] have been reported for growing cells to high densities. Among these, fed-batch is the most commonly used method to produce recombinant proteins [44].

**Figure 3:**
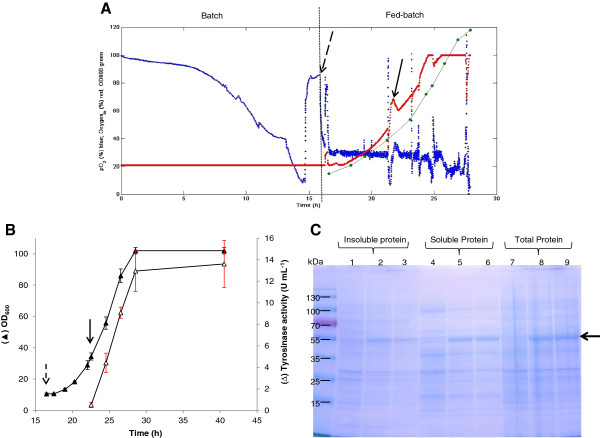
**Tyrosinase production of freshly transformed *E. coli *JM109 (pMFvpt) during the fed-batch cultivation.** Cells were grown at 32°C and induced with 1.25 mM IPTG at an OD_600_ value of 34. (**A**) Example of the fed-batch process applied in this study. The time course of the dissolved oxygen (pO_2_) (blue color), pure oxygen input (red color) and the OD_600_ values (green color) is shown. The dashed arrow indicates the time when feeding started; the solid arrow indicates the onset of induction. (**B**) Time-profiles of cell density (OD_600_) and volumetric activity (U mL^-1^) of tyrosinase during the fed-batch cultivation. The dashed arrow indicates the time when feeding started, the solid arrow indicates the onset of induction. (**C**) SDS-PAGE of proteins prepared from extracts of *E. coli* JM109 (pMFvpt). Samples were taken after 22 h just before induction (lanes 1, 4 and 7), after 28 h (6 h induction, lanes 2, 5 and 8), and after 40 h (18 h induction, lanes 3, 6 and 9). Lanes 1–3, insoluble proteins; Lanes 4–6, soluble proteins; lanes 7–9, total proteins. The arrow indicates the position of tyrosinase in the gel. Molecular weight standards are shown on the left.

Using exponential feeding would allow the culture to be kept in an extended exponential growth phase even at high cell density. In our case, production of recombinant tyrosinase is correlated with cell density during the growth (Figure 1). It was therefore decided to apply an exponential forward-feed strategy. Experiments were performed in a 1 L controlled bioreactor. Cells were first grown in batch medium at the optimal temperature of 32°C with glycerol as the carbon source. Batch growth, which preceded the fed-batch cultivation, resulted in a maximum growth rate of 0.4 h^−1^. Feeding started after 16 h of batch growth when glycerol was completely consumed (Figure 3A). The exponential mass-flow rate of glycerol in the feeding solution was set at 0.3 h^−1^. When OD_600_ reached about 34 (at 22 h of cultivation), cells were induced with 1.25 mM IPTG, and tyrosinase activity was followed with time. Figure 3B shows the growth profile and the total tyrosinase activity as a function of time during the fed-batch phase. The culture appeared to grow with a growth rate of 0.28 h^−1^. The difference between the feeding rate of 0.3 h^−1^ and the growth rate of 0.28 h^−1^ can be explained by the fact that glycerol was not only used for growth, but also for cell maintenance. It was also observed that when the feeding was stopped manually on purpose, the dissolved oxygen concentration instantly increased sharply (Figure 3A). This suggests that during the fed-batch mode the growing cells immediately consumed any glycerol that was added to the culture. The exponential growth phase lasted until the cell density reached 37 g L^-1^ (OD_600_ of 102) after 28 h of incubation, after which there was very little change in the cell density (Figure 3B). This was mainly due to the limitation of the bioreactor capacity: the dissolved oxygen could not be further increased even by using pure oxygen. Production of active tyrosinase increased steadily from 0 to 13 U mL^-1^ after 6 h induction, and increased only slightly from 13 to 13.6 U mL^-1^ with the prolongation from 28 to 40 h (Figure 3B). The obtained activity of 13 U mL^-1^ corresponded to 2.89 g L^-1^ of active tyrosinase in the culture based on our previous findings that the specific activity of purified tyrosinase is about 4.5 U mg^-1^[38]. Compared with the previously reported process where 1 g L^-1^ tyrosinase was produced in 6 days [36], the process established in this study allowed production of almost 3 g L^-1^ active tyrosinase in 28 hours, which is about 15 fold higher with respect to process productivity.

The optimal setting of the value for the feeding rate plays an important role in obtaining long-lasting fed-batch growth of *E. coli* cells. The feeding rate should maintain optimum cellular health, which is required to overcome the metabolic stress associated with recombinant protein expression [48]. A too high feeding rate would lead to accumulation of glycerol very early during the fed-batch, a premature slowdown of growth and, therefore, a short cultivation time. A too low feeding rate may lead to prolonged cultivation time before reaching high cell density, thus resulting in a low process productivity. It has been reported that a specific growth rate of 0.3 h^−1^ prevents several negative effects, such as increased cell lysis, higher levels of endotoxin accumulation and membrane stiffness, which are characteristics of cells at low specific growth rates [49,50]. An exponential feeding strategy to maintain a specific growth rate of 0.3 h^−1^ was therefore used in this study. A feeding rate of 0.4 h^−1^ was also tested and resulted in overfeeding and foaming problems (data not shown).

The tyrosinase content was further analyzed by SDS-PAGE (Figure 3C). The soluble tyrosinase was clearly predominant in the total soluble protein fraction after 6 h of induction and was maintained for at least another 12 h (Figure 3C). Compared to the batch cultures in shake flasks, the fed-batch approach resulted in an improvement of 17 fold of the yield of active tyrosinase from 0.17 g L^−1^ to 2.89 g L^−1^, and 15 fold of the overall volumetric productivity from 31.7 mU L^−1^ h^-1^ (corresponding to 7.1 mg L^−1^ h^−1^) to 464.3 mU L^−1^ h^-1^ (corresponding to 103.2 mg L^−1^ h^−1^) (Table 4). These results demonstrate that it was possible to obtain a high tyrosinase production even at high cell densities.

**Table 4:** Comparison of tyrosinase production in batch and fed-batch cultures

	**Cultivation time (h)**	**OD**_**max**_	**Vol. activity of tyrosinase (U mL**^**-1**^**)**	**Yield of tyrosinase (g L**^**-1**^**)**	**Vol. productivity of tyrosinase (mg L**^**-1**^ **h**^**-1**^**)**	**Vol. act. of tyrosinase per time (mU mL**^**-1**^ **h**^**-1**^**)**
Shake flask (Batch)	24	10.0 ± 0.9	0.76 ± 0.06	0.17 ± 0.01	7.1 ± 0.4	31.7 ± 2.5
Bioreactor (Fed-batch)	28	102.0 ± 2.0	13.00 ± 2.18	2.89 ± 0.48	103.2 ± 17.1	464.3 ± 77.8

No significant change in activity was observed even 12 h after the highest cell density in the fed batch experiment was reached (Figure 3B). Previously it has been observed that loss of tyrosinase activity due to proteolytic activity in recombinant *Streptomyces* was detected during all phases of batch culture, especially in stationary phase [51]. When the tyrosinase of *S. antibioticus* was expressed in *E. coli*, the activity of intracellular tyrosinase decreased with time [52]. The results obtained in this study suggest that *V. spinosum* tyrosinase produced in *E. coli* is stable, at least during the 40 hour cultivation tested here. This will undoubtedly simplify the purification of this enzyme.

### Stability of tyrosinase expression

**Figure 4:**
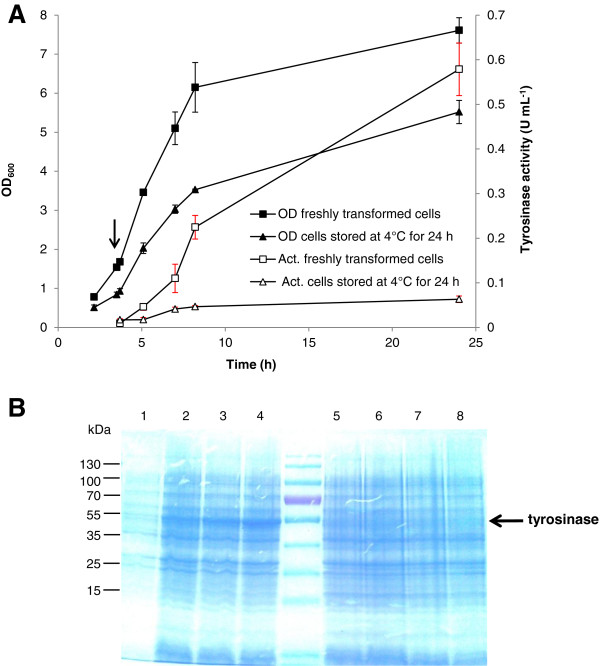
**Loss of tyrosinase expression in *E. coli *JM109 (pMFvpt).** The cells were grown in shake flasks at 32°C and induced with 1.0 mM IPTG. (**A**) Time profile of cell growth and tyrosinase activity of *E. coli* JM109 freshly transformed with pMFvpt and *E. coli* JM109 (pMFvpt) stored at 4°C for 24 h. The arrow indicates the onset of IPTG induction. (**B**) SDS-PAGE of total proteins prepared from *E. coli* JM109 (pMFvpt) cells. Samples were taken after 4 h just before induction (lanes 1 and 5), after 7 h (3 h induction, lanes 2 and 6), after 8 h (4 h induction, lanes 3 and 7), and after 24 h (20 h induction, lanes 4 and 8). Lanes 1–4, samples of freshly transformed *E. coli* JM109 (pMFvpt); Lanes 5–8, samples of 24 h old *E. coli* JM109 (pMFvpt). The arrow indicates the position of tyrosinase in the gel. Molecular weight standards are shown on the left.

During this study, we encountered loss of tyrosinase gene expression: when the starting inoculum was taken from a single colony on a plate which was stored at 4°C for more than 24 h, or from −80°C frozen stock, the recombinant cells lost the ability to produce tyrosinase under the tested conditions (Figure 4A). The cells were analyzed by SDS-PAGE and at the same time used for plasmid preparation. No tyrosinase band could be detected on the SDS-Gel (Figure 4B), even though resistance towards ampicillin conferred by the plasmid pMFvpt was detected in more than 95% of all cells (data not shown). For comparison, the purified plasmid was transformed freshly into *E. coli* JM109 cells and the resulting recombinant was used for tyrosinase production. In this case, tyrosinase production and activity were restored (Figures 4A and 4B). We have no proven explanation for this phenomenon. It is possible that the tyrosinase gene on the plasmid pMFvpt was lost. In order to obtain good expression of tyrosinase, the plasmid pMFvpt was always transformed freshly into *E. coli* JM109 cells during the entire study.

## Conclusion

In conclusion, we have successfully expressed and produced gram quantities per liter of active tyrosinase in recombinant *E. coli* by optimizing the expression conditions and fed-batch cultivation strategy. Fed-batch feeding was performed by exponential forward-feed mode which has the potential of reducing the fermentation time and thus cost. The recommended fermentation protocol is summarized in Table 5. In this protocol, exponential feed of substrate helped to increase the exponential phase of growth, thereby allowing a high cell density of 37 g L^-1^. This in turn helped in achieving a final concentration of about 3 g L^-1^ of soluble, active recombinant tyrosinase, which is 17 fold higher than that achieved in shake flasks. A specific tyrosinase production rate of 103 mg L^−1^ h^−1^ and a maximum volumetric activity of 464 mU L^−1^ h^-1^ were achieved in this study. These levels have not been reported previously.

**Table 5:** Summary of the optimized process used in this study for tyrosinase production

**Parameters**	**Tyrosinase production**
Strain	JM109 (pMFvpt)
Medium	Minimal medium containing glycerol and NZ-amine
Temperature (°C)	32
pH	6.9
Dissolved oxygen (%)	30
Cultivation	Fed-batch culture with exponential feeding
Feeding medium	Glycerol and NZ-amine
Feeding rate (h^-1^)	0.3
Induction stage	At OD_600_ value of 30-40
Harvest	End of the exponential growth

Since tyrosinase has potential for a broad range of applications, it is expected that an efficient production process will facilitate its actual usage. This work demonstrated an effective process of a bacterial tyrosinase production in the laboratory scale. Further improvement such as in the yield of active enzyme and validation of the scalability of the process are still needed.

## Methods

### Bacterial strains and plasmid

*E. coli* DH5α (*E*. *coli* Genetic Stock Center), BLR (Novagen) and JM109 (New England BioLabs) were tested as hosts for tyrosinase production. The plasmid pMFvpt, which contains the gene encoding the cytoplasmic full-length tyrosinase (53.5 kDa) of *V. spinosum*, was used to produce the recombinant tyrosinase [38].

### Materials

All chemicals used were purchased from Sigma–Aldrich (Buchs Switzerland) unless otherwise stated.

### Media

Luria broth (LB), 5 g yeast extract, 10 g tryptone, and 5 g NaCl per liter, was used for pre-inoculum cultures. It was supplemented with ampicillin to a final concentration of 0.1 mg mL^-1^.

The medium for batch cultures in shake flasks contained (g L^-1^): glycerol 10, NZ-amine 5, (NH_4_)_2_HPO_4_ 4, KH_2_PO_4_ 5, K_2_HPO_4_ 7.4, MgSO_4 *_ 7H_2_O 1.2, thiamine HCl 0.015, ampicillin 0.1 and 10 mL of trace element solution (TES). TES contained (g l^-1^): CaCl_2 *_ 2H_2_O 5, FeCl_3*_4H_2_O 7, Zn(CH_3_COO)_2 *_ 2H_2_O 1.3, MnCl_2 *_ 4H_2_O 1.5, CoCl_2 *_ 6H_2_O 0.25, H_3_BO_3_ 0.3, Na_2_MoO_4 *_ 2H_2_O 0.25, ethylenediaminetetraacetic acid (EDTA) 1.25 and 10 mL of concentrated HCl. Cells were induced with isopropyl β-D-1-thiogalactopyranoside (IPTG) under the conditions described in the Results section. Thiamine, ampicillin, TES and IPTG were filter-sterilized (0.2 μm, Millipore).

The medium for batch cultures in the bioreactor contained (g L^-1^): glycerol 20, NZ-Amine 5, (NH_4_)_2_HPO_4_ 4, KH_2_PO_4_ 13.3, (NH_4_)_2_SO_4_ 1, MgSO_4 *_ 7H_2_O 1.2, thiamine HCl 0.015, ampicillin 0.1 and 10 ml of TES containing additional 0.15 g l^-1^ CuCl_2 *_ 4H_2_O. The feed medium contained (g L^-1^): glycerol 500, NZ-Amine 100, MgSO_4 *_ 7H_2_O 13.5 and (NH_4_)_2_SO_4_ 50.

### Cultivation conditions

Plasmid pMFvpt was transformed into *E. coli* competent cells by chemical CaCl_2_ method [53]. The freshly transformed cells were used to inoculate a 10 mL LB pre-culture in a 50 mL flask. The cells were incubated at 37°C and 150 rpm overnight. The pre-culture was then used to inoculate 200 mL of batch medium in a 1 L shake flask with a dilution of 1:20 (v/v). It was incubated at 150 rpm and different temperatures as described in the Results section. Growth and product formation were monitored by periodically taking samples, from which cells were harvested by centrifugation at 12’000 g for 2 min at 4°C. A final OD_600_ of 7–8 was routinely achieved.

For the experiment of testing oxygen influence, 1 L baffled (with 4 baffles indented into the base, Schott Duran) and non-baffled Erlenmeyer flasks (Schott Duran) were used.

For bioreactor experiments, the cells freshly transformed with plasmid pMFvpt were grown in shake flasks in LB medium for 12 h at 37°C and 150 rpm. The bioreactors were inoculated with a 1:20 (v/v) dilution of the pre-culture. The cells were grown in a 600 mL (total volume 1.4 L) computer controlled bioreactor (Infors AG, Bottmingen, CH) equipped with standard control units. Tyrosinase expression was induced by 1.25 mM IPTG. The pH was maintained at 6.90 ± 0.05 by proportional–integral (PI) controlled addition of 4 M NaOH / 28% NH_4_OH 1 / 1 (v/v), and the temperature was set to 32.0°C ± 0.5°C. In order to avoid oxygen limitation the dissolved oxygen (DO) level was stabilized to above 30% saturation by stirrer speed and aeration rate control. If necessary, 1’200 rpm (rotation per minute) and 1–2 vvm (volume per volume per minute) of air enriched with pure oxygen by a PI controller were applied to keep the DO above 30%. Foaming was suppressed by addition of 10–100 μl antifoam suspension (PPG 2000). Feeding was started after the batch ended, as indicated by a sudden increase of the DO-signal. The feed medium was fed exponentially into the fermenter using a variable speed peristaltic pump.

### Tyrosinase activity

Samples were diluted to OD_600_ = 1, and 1 ml was centrifuged at 12’000 g for 2 min. The pellet was washed once with 1 ml and resuspended in 0.5 ml 0.1 M Tris–HCl (pH 8). The suspension was sonicated for 10 s with 10% power (Branson Ultrasonics Corp., Danbury, CT, USA), and centrifuged at 12’000 g for 2 min. The supernatant was analyzed for tyrosinase activity by measuring dopachrome formation [38,54]. One unit is defined as 1 μmol dopachrome formed per minute. The protein concentrations in the supernatant were quantified using the Bradford method.

### SDS-PAGE

SDS-PAGE analysis was performed using standard methods [55] and gels were subsequently stained with Coomassie brilliant blue. The PageRuler plus prestained protein ladder (Fermentas, GmbH) was used as a marker in all gels.

### Cell growth

The cell growth was followed by measuring the optical density at 600 nm (Spectronic Genesys 6, Thermo Electron Corp., UK) and correlated to cell dry weight (cdw) with a ratio cdw / OD = 0.36.

### Reproducibility

All measurements for growth and tyrosinase activity were performed at least in duplicates. The data presented in this report are the average values.

## Competing interests

The authors declare that they have no competing interests.

## Authors’ contributions

QR participated in the design and coordination of the study, and drafted the manuscript. BH participated in the design of the fed-batch study and performed the batch and fed-batch experiments. MF carried out the construction of different *E. coli* recombinants. LTM conceived the study and helped to draft the manuscript. All authors read and approved the final manuscript.
